# Pulmonary and Cardiorenal Cyclooxygenase-1 (COX-1), -2 (COX-2), and Microsomal Prostaglandin E Synthase-1 (mPGES-1) and -2 (mPGES-2) Expression in a Hypertension Model

**DOI:** 10.1155/2007/85091

**Published:** 2007-05-20

**Authors:** Zaher A. Radi, Robert Ostroski

**Affiliations:** ^1^Drug Safety Research & Development, Pfizer Global Research and Development, 2800 Plymouth Road, Building 50-G0503, Ann Arbor, MI 48105, USA; ^2^Department of Cardiovascular Pharmacology, Pfizer Global Research and Development, 2800 Plymouth Road, Building 50-G0503, Ann Arbor, MI 48105, USA

## Abstract

Hypertensive mice that express the human renin and angiotensinogen genes are used as a model for human hypertension because they develop hypertension secondary to increased renin-angiotensin system activity. Our study investigated the cellular localization and distribution of COX-1, COX-2, mPGES-1, and mPGES-2 in organ tissues from a mouse model of human hypertension. Male (*n* = 15) and female (*n* = 15) double transgenic mice (h-Ang 204/1 h-Ren 9) were used in the study. Lung, kidney, and heart tissues were obtained from mice at necropsy and fixed in 10% neutral buffered formalin followed by embedding in paraffin wax. Cut sections were stained immunohistochemically with antibodies to COX-1, COX-2, mPGES-1, and mPGES-2 and analyzed by light microscopy. Renal expression of COX-1 was the highest in the distal convoluted tubules, cortical collecting ducts, and medullary collecting ducts; while proximal convoluted tubules lacked COX-1 expression. Bronchial and bronchiolar epithelial cells, alveolar macrophages, and cardiac vascular endothelial cells also had strong COX-1 expression, with other renal, pulmonary, or cardiac microanatomic locations having mild-to-moderate expression. mPGES-2 expression was strong in the bronchial and bronchiolar epithelial cells, mild to moderate in various renal microanatomic locations, and absent in cardiac tissues. COX-2 expression was strong in the proximal and distal convoluted tubules, alveolar macrophages, and bronchial and bronchiolar epithelial cells. Marked mPGES-1 was present only in bronchial and bronchiolar epithelial cells; while mild-to-moderate expression was present in other pulmonary, renal, or cardiac microanatomic locations. Expression of these molecules was similar between males and females. Our work suggests that in hypertensive mice, there are (a) significant microanatomic variations in the pulmonary, renal, and cardiac distribution and cellular localization of COX-1, COX-2, mPGES-1, and mPGES-2, and (b) no differences in expression between genders.

## 1. INTRODUCTION

The renin-angiotensin-aldosterone system (RAAS) plays an important role in the control of cardiovascular and renal homeostasis by regulating vascular tone, blood pressure (BP), and fluid volume [1, 2]. Angiotensin II (Ang II) is a physiologically active component of the RAAS, produced via an enzymatic cascade that begins with angiotensinogen (AGT) cleaving renin (REN) to form angiotensin I (Ang I), which is then cleaved by the angiotensin converting enzyme (ACE) to form Ang II [3]. Ang II causes vasoconstriction directly by activating Ang II type 1 (AT1) receptors on vascular smooth muscle, affects fluid volume via AT1 receptor activation in the proximal tubule, resulting in renal sodium and water reabsorption, and plays an important role in the regulation of fluid balance by stimulating aldosterone secretion from the zona glomeruloza of the adrenal glands [3]. ACE inhibitors, Ang II receptor antagonists, and aldosterone receptor antagonists have been used as therapeutic interventions to treat hypertension.

The genes of the renin-angiotensin have been linked to and/or associated with hypertension in animal models and humans [2]. Recently, transgenic rodent models have been developed that over express both human REN and angiotensinogen, which leads to hypertension via chronic overproduction of Ang II. Specific examples include the murine double transgenic line (Ang 204/1 Ren 9), which produces a mean arterial BP 40 mmHg higher than background mice (C57Bl/6J) that lack the human genes [2]. These mice also had elevated aldosterone levels. In addition, transgenic rats harboring the mouse renin-2 gene developed hypertension, cardiac hypertrophy, and renal damage [4]. The Tsukuba hypertensive mice (THM), which express the human REN and angiotensinogen genes, have been proven to develop hypertension [5].

Originally, the RAAS was viewed solely as an endocrine system, in which angiotensinogen of hepatic origin is secreted intothe systemic circulation and cleaved by REN and ACE to produce the active peptide Ang II. However, there is increasing evidence that suggests a RAAS may reside within several organs or tissues, including kidney, lung, heart, and vascular smoothmuscle cells (SMC), where it is believed to act in a functionally independent paracrine/autocrine fashion [6]. This hypothesis is further supported by the fact that all components of the RAAS in the heart, kidney, and lung contain the ACE component [3, 6]. Additionally, high concentrations of Ang II have been demonstrated in the plasma, heart, and kidney of THM [7, 8].

In the kidney, prostaglandins (PGs) are important mediators of hemodynamic regulation, salt and water homeostasis, and REN release [9, 10]. The main PG in the kidney is PGE_2_, which is synthesized from arachidonic acid (AA) by enzymatic reactions, particularly cyclooxygenases and prostaglandin E synthases (PGES). Cyclooxygenase (COX) derived PGs have two distinct membrane-anchored isoenzymes, COX-1 and COX-2. COX-1 is constitutively expressed and found in most normal body tissues, while COX-2 is expressed in normal tissues at low levels and is highly induced by proinflammatory mediators in inflammation, injury, and pain settings [9]. The membrane-associated PGES-1 (mPGES-1) is inducible and functionally linked to COX-2, while mPGES-2 is constitutive and coupled to both COX isoforms [11].

It has been suggested that regulation of COX-2 in the kidney is altered by the RAAS system [9, 12]. In THM mice, increased expression of COX-2 in the macula densa has been reported [13], and an important role for RAAS in cardiac hypertrophy has been noted [14]. In addition, activation of RAAS has been demonstrated in rats with heart failure [6]. Overexpression of COX-2 has also been observed in the aldosterone-treated animals in normotensive and hypertensive rats [15].

In the lung, significant reduction in BP was seen in PG EP1 receptor-deficient mice and was accompanied by increased REN-Ang activity [16]. In addition, plasma Ang II levels were raised in severe asthma [17].

There are currently no published reports on the cellular expression and microanatomic location of COX-1, COX-2, mPGES-1, mPGES-2 in hypertensive transgenic mice. Therefore, using immunohistochemistry, we investigated the microanatomic location and cellular expression of COX-1, COX-2, mPGES-1, mPGES-2 in kidney, lung, and heart tissues obtained from renin-angiotensingen transgenic mice. This study is the first to report on the pulmonary and Cardiorenal microanatomic expression of these molecules in this animal model for human hypertension.

## 2. MATERIALS AND METHODS

### 2.1. Animals

15- to 20-week-old male (*n* = 15) and female (*n* = 15) double transgenic mice (h-Ang 204/1 h-Ren 9) were used in the study. The mice were derived from a founder colony of 5 male mice expressing human angiotensinogen (h-Ang 204/1) and 6 females expressing human renin (h-Ren6), obtained from Dr. Curt Sigmund at the University of Iowa, School of Medicine. At Charles River Laboratories (Wilmington, Massachusetts), female mice that expressed human REN were bred with angiotensinogen-expressing males to produce the double transgenic line. The transgenic line was developed on a C57/BI6J background. All the procedures were in compliance with the Pfizer Ann Arbor Laboratories Animal Care and Use Committee.

### 2.2. Study tissue samples

Lung, kidney, and heart tissues were obtained from the mice at the time of necropsy. Tissues were fixed in 10% neutral buffered formalin for 24 hours and embedded in paraffin wax. 3*μ*m-thick sections were then cut and stained immunohistochemically with antibodies to COX-1, COX-2, mPGES-1, and mPGES-2.

### 2.3. Immunohistochemistry for COX-1, COX-2, mPGES-1, and mPGES-2

To analyze the expression of COX-1, COX-2, mPGES-1, and mPGES-2, 3*μ*m sections were cut from formalin-fixed, paraffin-embedded blocks, mounted on positively charged glass slides, dried, and then loaded on the automated immunostainer (room temperature using a Ventana Discovery (Ventana Medical Systems, Tucson, AZ). Slides were deparaffinized and then rehydrated. Sections were incubated for 30 minutes with serum free DakoCytomation protein blocker (Dako Corporation, Dako, CA) and then rinsed and incubated for 4 minutes with Avidin-Biotin blocking solution (Ventana Medical Systems). Antigen retrieval was completed with a Ventana specialty solution (8 = pH) (Ventana Medical Systems).

Automation included exposure to 100*μ*L of primary anti-COX-1 (1:200), COX-2 (1:20), mPGES-1 (1:750), or mPGES-2 (1:1000) antibody (Cayman Chemical, Ann Arbor, MI) diluted with reagent diluent (Ventana Medical Systems) at room temperature for 60 minutes. 100*μ*L of the appropriate anti-rabbit biotinylated IgG linking solution (Vector Laboratories) was applied to each section at 1:200 dilution for 60 minutes at room temperature. Sections were again rinsed and allowed to react with 100*μ*L of diaminobenzidine (DAB detection kit) substrate solution (Ventana Medical Systems) for 8 minutes, followed by counterstaining with Hematoxylin and then Bluing Reagent for 4 minutes each, removed from the autostainer, washed in warm water, dehydrated through graded alcohol, cleared in xylene, and cover slipped. Control reactions included (1) sections incubated with the omission of primary antibody and processed as mentioned above, and (2) sections incubated with normal rabbit serum instead of the primary antibody and processed as above.

## 3. RESULTS

The pulmonary and Cardiorenal cellular expression and distribution of COX-1, COX-2, mPGES-1, and mPGES-2 are summarized in Tables 1, 2, 3, and 4. Staining intensity ranged from negative (−) to strong (+++).

**Figure 1:**
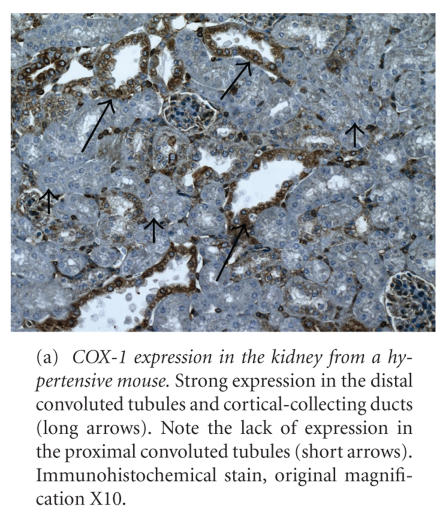

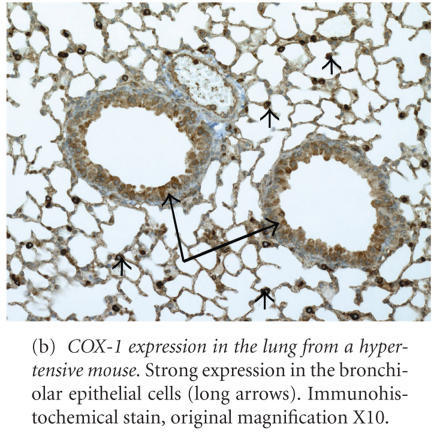


In the kidney, strong diffuse cytoplasmic COX-1 expression occurred in the distal convoluted tubules (DCT), cortical collecting ducts, and medullary collecting ducts, while proximal convoluted tubules (PCT) lacked COX-1 expression (see Figure 1(a) and Table 1). Moderate (++) diffuse COX-1 cytoplasmic staining was present in vascular endothelial cells (EC) and SMC, cortical interstitial cells (IC), the glomerular visceral epithelium, and the capsular parietal epithelium. Mild (+) COX-1 expression was present in medullary IC, glomerular podocytes, and the medullary ascending limb (MAL). Expression of COX-1 was equivocal in the macula densa.

In the lungs and heart, COX-1 was strongly expressed in alveolar macrophages, the bronchial and bronchiolar epithelium, and cardiac vascular endothelial cells, but moderately expressed in alveolar septa, bronchial smooth muscle cells, pulmonary vascular endothelial cells, pulmonary vascular EC, and cardiac vascular SMC. COX-1 was not expressed in cardiac myocytes (see Figure 1(b) and Table 2).

**Figure 2:**
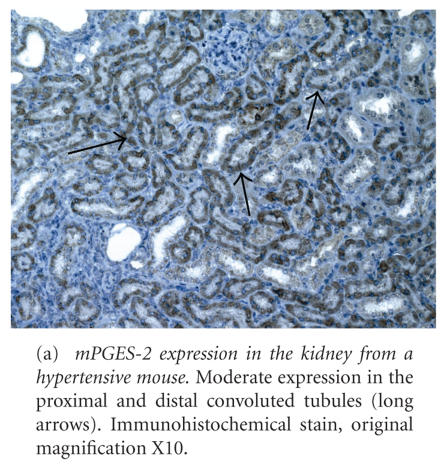

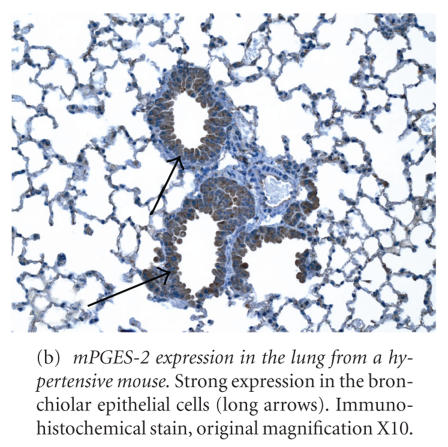


mPGES-2 was expressed in the kidney at a moderate diffuse level in PCT and DCT, capsular parietal epithelium, and medullary collecting ducts (see Figure 2(a) and Table 1). Mild expression was present in macula densa, vascular SMC and EC, glomerular podocytes and the visceral epithelium, cortical collecting ducts, and the MAL. No mPGES-2 expression was present in cortical or medullary IC.

Pulmonary expression of mPGES-2 included strong diffuse staining in bronchial and bronchiolar epithelial cells (see Figure 2(b) and Table 2), moderate staining in alveolar macrophages and septa, mild staining in bronchial SMC, and pulmonary vascular EC and SMC. No mPGES-2 expression was present in any of the cardiac microanatomic locations examined.

**Figure 3:**
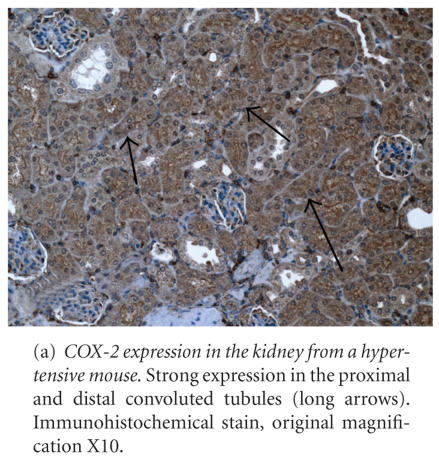

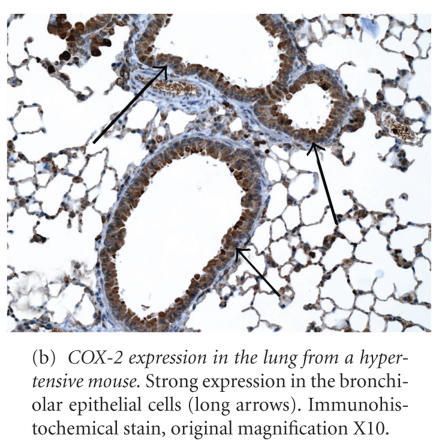


Renal COX-2 expression was strong in the PCT and DCT (see Figure 3(a) and Table 3), moderate in macula densa, vascular SMC and EC, and medullary collecting ducts, and mild in cortical and medullary interstitial cells, cortical collecting ducts, and the MAL. No expression was present in glomeruli or the capsular (parietal) epithelium.

In the lung, marked (+++) COX-2 expression was present in alveolar macrophages, and the bronchial and bronchiolar epithelium (see Figure 3(b) and Table 4). Other pulmonary microanatomic locations had mild or equivocal COX-2 expression. Cardiac vascular EC had mild COX-2 expression, while cardiac myocytes and cardiac vascular SMC were negative.

**Figure 4:**
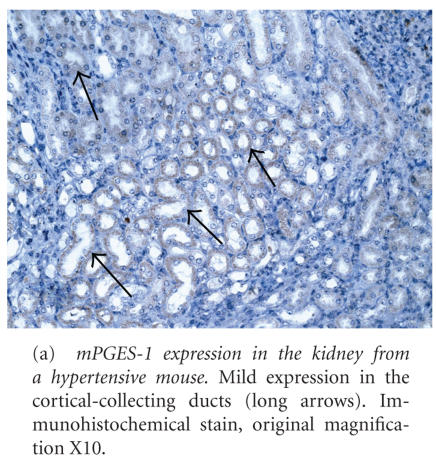

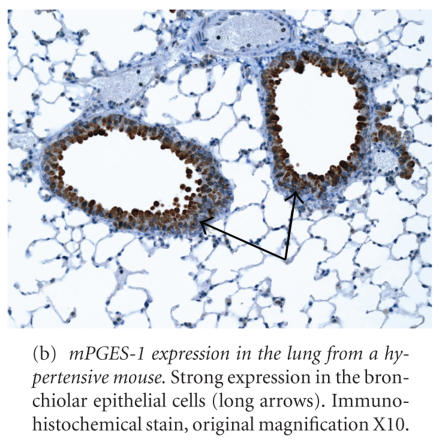


For mPGES-1, expression was mild in renal vascular SMC and EC, cortical and medullary collecting ducts, and the MAL (see Figure 4(a) and Table 3). Expression in the macula densa, glomerular (visceral) epithelium, and capsular (parietal) epithelium was equivocal. No mPGES-1 expression was present in other microanatomic renal locations.

mPGES-1 was the highest in bronchial and bronchiolar epithelial cells (see Figure 4(b) and Table 4). Moderate expression was present in bronchial SMC. Mild expression was present in alveolar macrophages and alveolar septa, while expression in pulmonary vascular EC and SMC was equivocal. Mild mPGES-1 expression was present in cardiac vascular EC and cardiac vascular SMC, while other cardiac microanatomic locations lacked expression. The expression of all these molecules was similar between males and females.

## 4. DISCUSSION

PGs are modulators of physiological functions and contribute to REN release, regulation of renal microvascular hemodynamics, salt balance, and BP controlvia mechanisms involving the regulation of vascular tone and renal excretory function. The kidney is capable of synthesizing all types of PGs, especially PGE_2_ and PGI_2_, which influence urinary sodium excretion directly through inhibition of the tubulartransport function and indirectly through the regulation of renin-angiotensin system activity [18]. COX, a rate-limiting enzyme in the PG biosynthesispathway, exists in two major isoforms: the constitutive COX-1 and the inducible COX-2 [19]. COX-1 is expressed constitutively at varying levels in the majority of tissues and generally plays a role in tissue homeostasis.

In this study, the highest degree of renal COX-1 expression occurred in the DCT, cortical collecting ducts, and medullary collecting ducts, while mild-to-moderate COX-1 expression occurred in other microanatomic renal locations. PCT lacked COX-1 expression. These results are concurrent with COX-1 being the most abundant COX isoform that is constitutively expressed in the kidney and regionally localized in the renal vasculature, collecting ducts, and papillary IC across various species [20, 21]. Further evidence supporting these results includes previous work in which various species (cow, dog, guinea pig, human, monkey, mouse, rabbit, rat, and sheep) exhibited high levels of COX-1 expression in the collecting ducts [20, 22, 23].

Since COX-1 is expressed in the collecting ducts of the nephron, an active area in the regulation of sodium excretion in both laboratory animals and humans [24], it is not surprising that BP increases in COX-1 deficient mice compared with wild-type controls [25]. Nonselective nonsteroidal anti-inflammatory drugs (NSAIDs) may aggravate renin-independent, sodium-sensitive hypertension, possibly in part by inhibition of the COX-1 responsible for sodium excretion [26]. In rats infused with selective COX inhibitors, NS-398 or meloxicam, direct renal interstitial volume expansion significantly increased renal interstitial hydrostatic pressure and fractional excretion of sodium [27].

Our finding that most cardiac microanatomic locations had COX-1 expression with the exception of cardiac myocytes is consistent with other studies showing COX-1 expression in both EC and SMC of the fetal ductus arteriosus of pigs, rats, sheep, and humans [28–31]. Furthermore, COX-1 was constitutively expressed in the EC of the aorta and microvasculature, fibrous connective tissue of the tricuspid valve, and chordae tendinae of the heart of dog [32].

All pulmonary microanatomic locations had COX-1 expression in our study. Other examples of COX-1 expression in lungs include human lungs [33], and the bronchiolar epithelium and smooth muscle, alveolar macrophages, EC, and vascular SMC of rat [34]. As a result, COX-1-dependent prostanoid generation has been implicated in the regulation of bronchial tone [34].

mPGES-2 is constitutively expressed in several tissues, is not induced during inflammation, and has been proposed to mediate PGE_2_ production from both COX isoforms [11, 35]. In our study, moderate mPGES-2 expression was present in PCT and DCT, the capsular parietal epithelium, and medullary collecting ducts, while mild or no expression was present in other renal microanatomic locations. In other studies, high expression of mPGES was reported in distal tubules, and medullary collecting ducts in normal mouse kidney [36]. mPGES-2 was expressed in all pulmonary microstructures examined.

In the heart, mPGES-2 was not present in any microanatomic locations. Other studies using primary cultures of neonatal ventricular myocytes have shown that mPGES-2 is constitutively synthesized in myocytes and is not regulated [37].

COX-2 is expressed in variable locations in the kidney across various species [20]. COX-2 deficient mice exhibited severe disruption of renal development and function, suggesting an important role for COX-2 in renal development [38, 39]. The normal rodent and canine kidney have prominent constitutive COX-2 expression in the macula densa and thick ascending limb of loop of Henle, whereas COX-2 is absent at these sites in the normal primate and human kidney [21]. In our study, COX-2 was present in all microanatomic renal locations, with the exception of the glomeruli and capsular (parietal) epithelium. This finding is consistent with the known regulatory association between COX-2 and REN reported by Schnermann [40]. Demonstration that indomethacin and SC58236 (a selective COX-2 inhibitor) can decrease BP and suppress plasma REN activity in rats lends further support to this association [41, 42]. Conversely, COX-2 knockout mice had reduced REN content and activity [43].

The marked increase of COX-2 expression in the pulmonary epithelium in our study is consistent with the known role of respiratory epithelia as a first line of defense. Pulmonary epithelial cells have an important role in airway homeostasis, perform many important biological functions, and represent the first line of defense against infection [44]. Activation of the renin-angiotensin system has also been demonstrated in acute asthma [17].

Cardiac vascular EC and SMC had COX-2 expression, while no expression was present in cardiac myocytes or SMC in our study. Ang II is known to stimulate vascular SMC growth [45]. An important role for Ang II in the regulation of COX-2 has been reported [46].

mPGES-1 is a source of inducible PGE_2_ synthetic activity [47]. In this study, mPGES-1 was present in renal vascular EC and SMC, cortical and medullary collecting ducts, MAL, all pulmonary microanatomic locations, and cardiac vascular EC and SMC, while no expression was present in other renal or cardiac microanatomic locations. These findings are consistent with increased mPGES expression described in patients with hyperprostaglandin E syndrome [48]. mPGES-1 was also induced by the proinflammatory cytokine in cardiac myocytes and fibroblasts [37].

In conclusion, in hypertensive mice there are (a) significant microanatomic variations in the pulmonary, renal, and cardiac distribution and cellular localization of mPGES-1, mPGES-2, COX-1, and COX-2 and (b) no differences in expression between genders.

## Figures and Tables

**Table 1:** Renal immunohistochemical expression of mPGES-2 and COX-1. PCT = proximal convoluted tubules; DCT = distal convoluted tubules; SMC = smooth muscle cells; EC = endothelial cells; IC = interstitial cells; MAL = medullary ascending limb.

Cellular location	mPGES-2	COX-1

Macula densa	+	±
PCT	++	−
DCT	++	+++
Vascular SMC	+	++
Vascular EC	+	++
Cortical IC	−	++
Medullary IC	−	+
Glomeruli (podocytes)	+	+
Glomerular (visceral) epithelium	+	++
Capsular (parietal) epithelium	++	++
Cortical collecting ducts	+	+++
Medullary collecting ducts	++	+++
MAL	+	+

(±) = equivocal staining; (−) = no staining, (+) = mild staining; (++) = moderate staining; (+++) = strong staining.

**Table 2:** Pulmonary and cardiac immunohistochemical expression of mPGES-2 and COX-1. SMC = smooth muscle cells; EC = endothelial cells.

Cellular location	mPGES-2	COX-1

Alveolar macrophages	++	+++
Alveolar septa	++	++
Bronchial epithelium	+++	+++
Bronchial SMC	+	++
Bronchiolar epithelium	+++	+++
Pulmonary vascular EC	+	++
Pulmonary vascular SMC	+	++
Cardiac myocytes	−	−
Cardiac vascular SMC	−	++
Cardiac vascular EC	−	+++

(−) = no staining; (+) = mild staining; (++) = moderate staining; (+++) = strong staining.

**Table 3:** Renal immunohistochemical expression of mPGES-1 and COX-2. PCT = proximal convoluted tubules; DCT = distal convoluted tubules; SMC = smooth muscle cells; EC = endothelial cells; IC = interstitial cells; MAL = medullary ascending limb.

Cellular location	mPGES-1	COX-2

Macula densa	±	++
PCT	−	+++
DCT	−	+++
Vascular SMC	+	++
Vascular EC	+	++
Cortical IC	−	+
Medullary IC	−	+
Glomeruli (podocytes)	−	−
Glomerular (visceral) epithelium	±	−
Capsular (parietal) epithelium	±	−
Cortical collecting ducts	+	+
Medullary collecting ducts	+	++
MAL	+	+

(±) = equivocal staining; (−) = no staining; (+) = mild staining; (++) = moderate staining; (+++) = strong staining.

**Table 4:** Pulmonary and cardiac immunohistochemical expression of mPGES-1 and COX-2. EC = endothelial cells; SMC = smooth muscle cells.

Cellular location	mPGES-1	COX-2

Alveolar macrophages	+	+++
Alveolar septa	+	+
Bronchial epithelium	+++	+++
Bronchial SMC	++	+
Bronchiolar epithelium	+++	+++
Pulmonary vascular EC	±	+
Pulmonary vascular SMC	±	±
Cardiac myocytes	−	−
Cardiac vascular SMC	+	−
Cardiac vascular EC	+	+

(±) = equivocal staining; (−) = no staining; (+) = mild staining; (++) = moderate staining; (+++) = strong staining.
